# *ABCB1* polymorphism in clopidogrel-treated Montenegrin patients

**DOI:** 10.1515/biol-2021-0017

**Published:** 2021-02-18

**Authors:** Snezana Mugosa, Zoran Todorovic, Jelena Cukic, Majda Sahman-Zaimovic, Natasa Djordjevic

**Affiliations:** Faculty of Medicine, Department of Pharmacology, University of Montenegro, 81104 Podgorica, Montenegro; Institute for Medicines and Medical Devices of Montenegro, 81104 Podgorica, Montenegro; Department of Pharmacology, Clinical Pharmacology Toxicology, Faculty of Medicine, University of Belgrade, University Medical Center “Bežanijska kosa”, 11000 Belgrade, Serbia; Department for Immunology and Virology, Public Health Institute, 34000 Kragujevac, Serbia; Department of Pharmacology and Toxicology, Faculty of Medical Sciences, University of Kragujevac, Svetozara Markovica 69, 34000 Kragujevac, Serbia

**Keywords:** *ABCB1*, P-glycoprotein, clopidogrel, single nucleotide polymorphism

## Abstract

Clopidogrel is an antiplatelet drug that displays significant interindividual variability in treatment response. Its bioavailability depends on the function of P-glycoprotein (P-gp), which is coded by a highly polymorphic *ABCB1* gene. Thus, the aim of this study was to investigate the effect of *ABCB1* genetic polymorphism on clopidogrel efficacy and safety and to determine the frequency distribution of its most common single nucleotide polymorphisms (SNPs) in 106 Montenegrin cardiology patients. Clopidogrel efficacy and safety were followed up during 1 year after hospitalization, with the lack of efficacy and adverse drug reactions observed in 11 (10.4%) and 8 patients (7.5%), respectively. Genotyping for *ABCB1* SNPs rs1128503 (1236C > T), rs2032582 (2677G > A/T), and rs1045642 (3435C > T) was performed by the real-time PCR method, and the variant alleles were detected with the frequencies of 42.9, 44.8, and 52.8%, respectively. No significant association was observed between any of the examined genotypes and clopidogrel efficacy (*p* = 0.253) or safety (*p* = 0.424). Due to small sample size, co-treatment with other drugs, and other genetic factors not taken into account, we believe the absence of correlation between *ABCB1* genotypes and indicators of clopidogrel efficacy and safety in this study should be apprehended conditionally, and that larger and better-controlled studies are warranted.

## 1 Introduction

Clopidogrel is an antiplatelet drug that inhibits platelet activation and aggregation by blocking adenosine diphosphate P2Y12 receptor [1]. Along with aspirin, it represents the mainstay in the management of patients suffering from myocardial infarction, ischemic stroke, acute coronary syndrome, and atrial fibrillation [2]. However, clopidogrel therapy is not always efficient or safe since the possible outcomes range from complete resistance to treatment to major bleeding events [3].

The observed interindividual variability in response to clopidogrel is multifactorial [4], but it mainly results from the heterogeneity of the achieved active drug plasma level [5]. Since clopidogrel is taken orally, its bioavailability depends on its absorption in the intestine and its metabolism in the liver, the former being partly regulated by an efflux transporter known as P-glycoprotein (P-gp) [6]. P-gp is encoded by a highly polymorphic *ABCB1* (ATP binding cassette subfamily B member 1, a.k.a. *MDR1*, i.e., multidrug resistance 1) gene [7], whose variations have been frequently, but not consistently, associated with altered overall P-gp transporting activity [8]. In the coding region of *ABCB1*, the most common and the most frequently studied single nucleotide polymorphisms (SNPs) are two synonymous and one non-synonymous variant, namely, 1236C > T, 2677G > A/T, and 3435C > T [8]. In spite of numerous investigations involving many P-gp substrates, the role of these SNPs in protein expression and functional integrity, as well as their importance for drug therapy, is still stirring up controversy. Contradictory results include studies that have reported lower P-gp expression, higher drug levels, or more pronounced effects in carriers of variant alleles [9], as opposed to others, where the observed effects were partly or entirely inverse [10], or not detected at all [11]. Clopidogrel studies yielded conflicting conclusions too, with observations of lower drug plasma concentration, impaired response to therapy, and a higher risk of bleeding in carriers of variant 3435T allele [12], counterbalanced by the opposite findings lacking any significant association of *ABCB1* polymorphism with clopidogrel pharmacokinetics or platelet response [13].

The frequency distribution of *ABCB1* SNPs displays pronounced interethnic variations [10], which could at least in part explain the discrepancies in the reported effects of *ABCB1* polymorphism. In Montenegro, clopidogrel is widely prescribed [14], but rarely followed through as recommended, usually due to increased risk of bleeding or the lack of therapeutic effects [4,15]. The importance of pharmacogenetic polymorphism for clopidogrel response in Montenegrins has already been reported [4]. However, as to our best knowledge, *ABCB1* variations have not been studied in this population. Thus, the main aim of this study was to investigate the frequency distribution of the most common *ABCB1* SNPs, and their potential effect on clopidogrel efficacy and safety, in Montenegrin cardiology patients.

## 2 Materials and methods

### 2.1 Study participants

The study involved 106 clopidogrel-treated inpatients from the Cardiology Department of the Clinical Centre of Montenegro, Podgorica. The exclusion criteria were age younger than 18 and older than 80, pregnancy or breastfeeding, and known contraindication for clopidogrel (including hypersensitivity, severe hepatic impairment, and active pathological bleeding). Clopidogrel was prescribed for approved indications (including acute coronary syndrome and atrial fibrillation) at 75 mg daily dose to all the patients. All the patients were co-prescribed with acetylsalicylic acid, which is not a substrate for P-gp [16].

**Figure A1:**
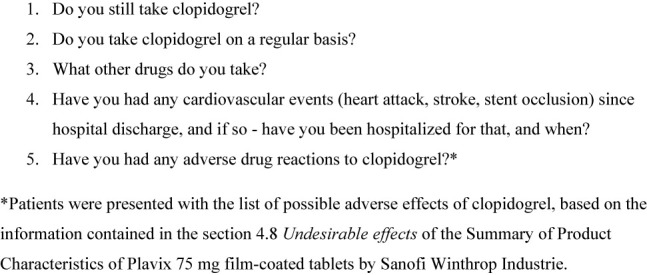
Questions included in patient interview 1 year after hospital discharge.

Clopidogrel treatment was considered efficient if acute coronary syndrome, stent thrombosis, stroke, percutaneous coronary intervention, or death did not occur within 1 year after initial hospitalization. Clopidogrel safety was assessed based on the occurrence of common adverse reactions, i.e., bleeding complications. The relevant data were collected through interviews and questionnaires during hospitalization and 1 year after hospital discharge (Figure A1). Patients who were deemed to be clopidogrel-resistant and experienced clopidogrel-related safety issues were considered cases, as opposed to controls, who reacted well to clopidogrel and had no adverse reactions, respectively.


**Informed consent:** Informed consent has been obtained from all individuals included in this study.
**Ethical approval:** The research related to human use has been complied with all the relevant national regulations, institutional policies, and in accordance with the tenets of the Helsinki Declaration and has been approved by the Ethics Committee at the Clinical Centre of Montenegro, Podgorica (approval No 03/01-14664/1).

### 2.2 *ABCB1* genotyping

DNA was extracted from the whole-blood EDTA samples using the Purelink™ genomic DNA kit (Invitrogen, Carlsbad, CA, USA). Genotyping for three common *ABCB1* SNPs, rs1128503 (1236C > T), rs2032582 (2677G > A/T), and rs1045642 (3435C > T), was performed on SaCycler-96 (Sacace Biotechnologies, Como, Italy) by the real-time PCR method, using TaqMan Genotyping Master Mix and the corresponding TaqMan DME genotyping assays (Thermo Fisher Scientific, Waltham, MA, USA).

### 2.3 Statistical analysis

*ABCB1* haplotype analysis and haplotype frequency calculations were performed using Arlequin, version 3.11 (http://cmpg.unibe.ch/software/arlequin3). Testing for Hardy–Weinberg equilibrium was performed with the *χ* ^2^ test. IBM SPSS Statistics, version 22 (IBM Corporation, Armonk, NY, USA), was employed in statistical analysis, with *p* < 0.05 considered significant. The influence of *ABCB1* genotype and concomitant drug therapy on efficacy and safety of clopidogrel treatment was assessed using the *χ* ^2^/Fisher exact test.

## 3 Results

The study subjects included 72 men and 34 women, 32 to 77 years old (mean ± SD: 59.5 ± 8.9), diagnosed with either myocardial infarction (*n* = 50), angina pectoris (*n* = 52), or atrial fibrillation (*n* = 4). Eighty-eight patients (83.0%) were co-treated with at least one drug known to either increase (tirofiban, warfarin, or acenocoumarol (*n* = 3), heparin or enoxaparin (*n* = 37)) or decrease its serum level and/or effect (six receiving felodipine, nifedipine, or diltiazem, and 81 omeprazole or pantoprazole). In addition, five patients were co-treated with other drugs that do not interact with clopidogrel, i.e., prednisone or methylprednisone. Clopidogrel was ineffective in 11 patients (10.4%), while in 8 cases (7.5%) it caused at least 1 bleeding episode as an adverse drug reaction.

The frequencies of the *ABCB1* SNPs, genotypes, haplotypes, and diplotypes are presented in Table 1. None of the SNPs deviated from Hardy–Weinberg equilibrium (*p* > 0.07). Comparison among *ABCB1* genotypes did not reveal any significant association of 1236C > T, 2677G > A/T, or 3435C > T with clopidogrel efficacy (*p* = 0.253). Similarly, no association was observed between any SNPs and bleeding episodes as the indicator of clopidogrel safety (*p* = 0.424). Concomitant therapy did not appear to affect clopidogrel efficacy (*p* = 0.244) or safety (*p* = 0.255).

**Table 1:** SNP, genotype, haplotype, and diplotype frequencies of *ABCB1* in Montenegrin clopidogrel-treated patients

			Observed frequency	95% Confidence interval
Allele	rs1128503 (1236C < T)		0.429 (91/212)	0.365, 0.497
	rs2032582 (2677G < T/A)		0.448 (95/212)	0.383, 0.515
	rs1045642 (3435C < T)		0.528 (112/212)	0.461, 0.594
Genotype	rs1128503 (1236C < T)	CC	0.302 (32/106)	0.223, 0.395
		CT	0.538 (57/106)	0.443, 0.630
		TT	0.160 (17/106)	0.102, 0.243
	rs2032582 (2677G < T/A)	GG	0.321 (34/106)	0.102, 0.243
		GT	0.462 (49/106)	0.370, 0.557
		GA	0.000 (0/106)	0.000, 0.043
		TT	0.208 (22/106)	0.141, 0.295
		AT	0.009 (1/106)	0.000, 0.058
		AA	0.000 (0/106)	0.000, 0.043
	rs1045642 (3435C < T)	CC	0.179 (19/106)	0.117, 0.264
		CT	0.585 (62/106)	0.490, 0.674
		TT	0.236 (25/106)	0.165, 0.326
Haplotype^a^	C-G-C		0.415 (88/212)	0.351, 0.482
	T-T-T		0.354 (75/212)	0.293, 0.420
	C-G-T		0.075 (16/212)	0.046, 0.120
	C-T-T		0.066 (14/212)	0.039, 0.109
	T-G-C		0.033 (7/212)	0.015, 0.068
	T-G-T		0.028 (6/212)	0.012, 0.062
	T-T-C		0.014 (3/212)	0.003, 0.043
	C-T-C		0.009 (2/212)	0.001, 0.036
	C-A-T		0.005 (1/212)	0.000, 0.029
Diplotype^a^	C-G-C/T-T-T		0.349 (37/106)	0.265, 0.444
	T-T-T/T-T-T		0.123 (13/106)	0.072, 0.200
	C-G-C/C-G-T		0.113 (12/106)	0.065, 0.190
	C-G-C/C-G-C		0.104 (11/106)	0.058, 0.179
	C-G-C/T-G-T		0.057 (6/106)	0.024, 0.121
	C-G-C/C-T-T		0.038 (4/106)	0.012, 0.097
	C-G-C/T-G-C		0.038 (4/106)	0.012, 0.097
	C-G-T/T-T-T		0.038 (4/106)	0.012, 0.097
	C-T-T/T-T-T		0.038 (4/106)	0.012, 0.097
	C-T-T/C-T-T		0.028 (3/106)	0.007, 0.084
	C-G-C/C-T-C		0.019 (2/106)	0.001, 0.071
	T-T-C/T-T-T		0.019 (2/106)	0.001, 0.071
	C-G-C/T-T-C		0.009 (1/106)	0.000, 0.058
	T-T-T/C-A-T		0.009 (1/106)	0.000, 0.058
	T-G-C/T-G-C		0.009 (1/106)	0.000, 0.058
	T-G-C/T-T-T		0.009 (1/106)	0.000, 0.058

a 1236C/T-2677G/T/A-3435C/T.

## 4 Discussion

Clopidogrel has unfavorable pharmacokinetics. Its bioavailability, which varies considerably, has been deemed essential for developing both drug resistance and bleeding episodes as its adverse effects. This is particularly important considering that usually only 10–15% of clopidogrel dose becomes active after administration, mainly due to biotransformation by *CYP2C19* [17]. We have previously shown the importance of *CYP2C19* genetic polymorphism for variability in therapeutic response to clopidogrel in Montenegrin cardiology patients [4]. Our previous findings remain in line with the Cochrane database and the Clinical Pharmacogenetics Implementation Consortium Guideline, recommending clinical use and confirming the cost-effectiveness of *CYP2C19* genotype testing in patients prescribed with clopidogrel [18,19].

However, clopidogrel is an orally administered drug whose bioavailability additionally depends on the rate of intestinal absorption, and this process seems to be significantly affected by intestinal expression and function of P-gp [6]. Thus, we have broadened our pharmacogenetic analysis of clopidogrel efficacy and safety as to include the potential role of most common and tenably functional SNPs of P-gp-coding gene *ABCB1*. As to our best knowledge, this is the first study related to *ABCB1* genetic polymorphism in Montenegrins. Although we did determine and report frequency distributions of *ABCB1* SNPs 1236C > T, 2677G > A/T, or 3435C > T in Montenegrin cardiology patients, we failed to detect any significant correlation between *ABCB1* genetic polymorphism and clopidogrel efficacy and safety.

The overall frequency distributions of 1236C > T, 2677G > A/T, and 3435C > T *ABCB1* variants display significant interethnic variability [8], with differences as high as 62 vs 13% of European Americans carrying variant alleles as compared to African Americans, respectively [10]. Similarly, comparison in terms of allele frequency between Africans, Caucasians, and Asians revealed marked variations for each of the alleles as well, e.g., 3435T frequency distribution spanning from 17% in Ghana and Kenya, up to 57% in Portugal and 66% in South-west Asia [20]. Yet, among Caucasians examined, *ABCB1* SNPs are rather uniformly distributed, 1236T ranging from 41% in German [21], 42% in French [22] to 46% in Serbian [23], 2677A/T from 45% in French [22] and German [21], 47% in Serbian [23] to 49% in Hungarians [24], and 3435T from 46% in French [22], 52% in British [20], 53% in Serbian [23], 54% in German [21], to 57% in Hungarians [24]. It is easy to note that *ABCB1* genetic polymorphism observed in Montenegrins conforms well to the previously published European data, including those from other clopidogrel-receiving patients [25,26,27].

It has been confirmed that the presence of *ABCB1* 3435C > T alters the pharmacokinetics of clopidogrel [6,28], as well as many other drugs [29]. As 3435C > T is a synonymous variant, which by definition is not expected to change primary structure of the coding protein [30], in an attempt to understand its role in drug transport other mechanisms should be considered. While its effect can be the result of altered messenger RNA splicing or P-gp protein stability, one of the most plausible explanations might actually be a strong linkage disequilibrium with other non-synonymous SNPs, including 1236C > T and 2677G > A/T. As the impact of *ABCB1* genetic polymorphism on clopidogrel pharmacokinetics requires comprehensive analysis, in this study we have estimated both separate and combined effects of all three SNPs, i.e., assessed the impact of both individual *ABCB1* SNPs and *ABCB1* 1236C/T-2677G/T/A-3435C/T genotypes on patients’ response to clopidogrel treatment.

Although no significant difference could be observed between carriers and noncarriers of specific alleles or genotypes in terms of clopidogrel efficacy and safety, it should be noted that the number of patients included in this study is relatively small, that the patients were not on monotherapy with clopidogrel but rather treated with other drugs with potential for both pharmacokinetics and pharmacodynamics interactions with clopidogrel, as well as that other already confirmed genetic factors [4] were not taken into account during the analysis. Thus, the absence of correlation between *ABCB1* genotypes and indicators of efficacy and safety of clopidogrel in this study should be apprehended conditionally, although it conforms well to conclusions of several similar studies [31,32,33,34,35]. Namely, previously published larger and better-controlled studies, which have observed higher risk of clopidogrel resistance [36,37] and higher risk of major cardiovascular adverse events [38] in carriers of different variant *ABCB1* alleles, warrant further investigation of the role of *ABCB1* genetic polymorphisms not only in Montenegrins but also in other populations, where clopidogrel remains one of the most frequently prescribed antiplatelet drugs.

## Figures and Tables

**Figure A2:**
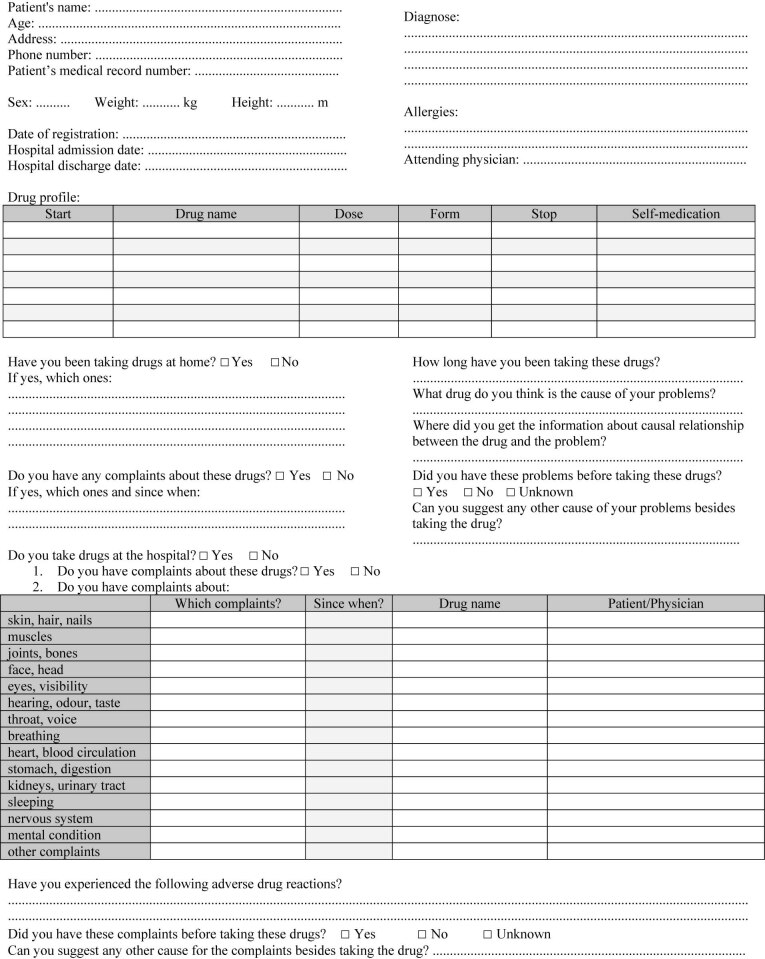
Patient questionnaire during hospitalization.
